# Early neutrophil count relates to infarct size and fatal outcome after large hemispheric infarction

**DOI:** 10.1111/cns.13381

**Published:** 2020-05-06

**Authors:** Li‐li Cui, Yan Zhang, Zhong‐yun Chen, Ying‐ying Su, Yawu Liu, Johannes Boltze

**Affiliations:** ^1^ Department of Neurology Xuanwu Hospital Capital Medical University Beijing China; ^2^ Department of Neurology and Clinical Radiology University of Eastern Finland Kuopio Finland; ^3^ School of Life Sciences University of Warwick Coventry UK

**Keywords:** brain edema, large hemispheric infarction, neutrophil, outcome

## Abstract

**Aims:**

To investigate the relationship between peripheral leukocyte dynamics and the outcome of large hemispheric infarction (LHI) patients.

**Methods:**

Patients with acute LHI admitted to the neuro‐intensive care unit of Xuanwu Hospital from 2013 to 2017 were prospectively enrolled and followed up for 6 months after LHI.

**Results:**

A total of 84 LHI patients were included, 38 patients suffered brain herniation and 20 patients died from stroke. Compared to patients with benign course, LHI patients with fatal outcome showed larger infarcts and more severe brain edema (*P* < .01), as well as increased WBC and neutrophil counts throughout the first week after stroke (*P* < .05). Correlation analysis revealed that neutrophil counts on D2 after LHI positively correlated with infarct and edema volumes measured from CT/MRI (*R^2^* = 0.22 and *R* ^2^ = 0.15, *P* < .01) and negatively correlated with Glasgow Coma Scale (ρ = −0.234, *P* < .05). Patients with D2 neutrophils > 7.14 × 10^9^/L had higher risk of brain herniation [odds ratio (OR) = 7.5, 95% CI: 2.0‐28.1, *P* = .001], and patients with D2 neutrophils > 7.79 × 10^9^/L had a higher risk of death (OR = 5.8, 95% CI: 1.2‐27.0, *P* = .015).

**Conclusion:**

Early peripheral neutrophil count after stroke relates to infarct size and the fatal outcome of LHI patients, which might help guiding acute LHI management such as reduction of intracranial pressure and potential antiinflammatory therapy in the future.

## 1 INTRODUCTION

Large hemispheric infarction (LHI) accounts for approximately 10% to 15% of ischemic stroke events and is associated with high mortality and poor outcome.^1^ Malignant brain edema is frequently seen in LHI, which could cause brain herniation and potentially death.^1^, ^2^ Brain herniation can hardly be reversed by medication alone, and timely decompressive craniectomy is often needed to reduce mortality.^3^, ^4^ It is thus important to identify indicators of malignant brain edema and potential brain herniation to prevent severe neurological dysfunction and death by early intervention.

Leukocytes, particularly neutrophils, have been reported to play an important role in experimental stroke.^5^, ^6^, ^7^ However, the evidence in stroke patients is still limited.^8^ Moreover, few studies have investigated the dynamics of leukocyte after LHI and its relationship with infarct and edema volumes, or fatal outcomes including brain herniation and death. Therefore, we aimed to investigate the potential relationship between leukocyte changes of LHI patient within one week after symptom onset and the outcome including brain herniation and death.

## 2 METHODS

This observational, prospective study was approved by the Ethics Committee of Xuanwu Hospital, Capital Medical University (No. 2008‐1) and complied with the Declaration of Helsinki.

### 2.1 Patient inclusion and exclusion criteria

Patients with acute LHI admitted to the neuro‐intensive care unit (NICU) of Xuanwu Hospital, Capital Medical University from January 2013 to December 2017 were enrolled in this study. Informed consents were obtained from patients or their relatives. LHI diagnosis and management were performed according to the established guidelines.^2^, ^9^ The inclusion criteria were (1) 18‐85 years old; (2) unilateral LHI involving at least 2/3 of the middle cerebral artery (MCA) territory, confirmed by computed tomography (CT) or magnetic resonance imaging (MRI); (3) within 48 hours after LHI onset; and (4) modified Rankin Scale (mRS)≤2 before symptom onset. Exclusion criteria were (1) comorbidities of serious organ dysfunction or cancer; (2) infection within 48h after symptom onset; (3) death due to reasons other than brain herniation; and (4) cerebral lobe hemorrhage > 30 mL or ventricular hemorrhage. Brain herniation (in particular uncal herniation) was defined by deterioration of consciousness, a failure of upper brain stem function, and CT or MRI findings including severe cerebral edema, cerebral ventricular, and brain stem compression with midline shift.^9^

### 2.2 Clinical assessment and data collection

Blood samples were collected on the 2nd (ie, 24h‐48h), 4th, and 7th day (D2, D4, and D7) after LHI onset for whole blood cell test, including white blood cell (WBC), neutrophil, and lymphocyte counts as well as neutrophil‐to‐lymphocyte ratio (NLR). Changes in comparison to D2 were shown as Δ. We also recorded (1) age; (2) sex; (3) history of smoking and drinking alcohol; (4) occurrence of headache, vomiting, disturbance of consciousness, and gaze palsy; (5) body temperature from D2 to D7; (6) blood pressure (BP), National Institutes of Health Stroke Scale (NIHSS), Glasgow Coma Scale (GCS), levels of procalcitonin (PCT), C‐reactive protein (CRP), neuron‐specific enolase (NSE), and blood glucose on D2 after symptom onset; (7) volume of cerebral infarction, which was measured semi‐automatically from head CT or diffusion‐weighted imaging (DWI) sequence of MRI on D2 after LHI using Fiji software and corrected with indirectly measured brain edema^10^; (8) stroke etiology according to the TOAST classification (large artery atherosclerosis, cardiogenic cerebral embolism, small‐artery occlusion, other determined and undetermined etiology)^11^; (8) history of atrial fibrillation, cardiac dysfunction, hypertension, and diabetes mellitus; and (9) thrombolysis, endovascular treatment, and/or decompressive craniectomy.^2^, ^9^ Patients were followed up for 6 months after symptom onset, and mRS at 6 months was recorded.

### 2.3 Statistical analysis

Statistical analyses were performed with SPSS 22.0 (IBM Corporation, Armonk, NY). Kolmogorov‐Smirnov test was used to check the normality of continuous data. Normally distributed data were expressed as mean ± standard deviation (SD), while the non‐normally distributed data were expressed as median (interquartile range, IQR). Student's t‐test, Mann‐Whitney U‐test, or chi‐square test was used for intergroup comparisons, when appropriate. Repeated measures ANOVA was used to explore the group‐time effects regarding the changes of peripheral leukocyte counts within the first week poststroke. Pearson's correlation analysis was performed to explore the correlation between the peripheral leukocyte count and infarct/edema volumes. Spearman's analysis was used to investigate the correlation between peripheral leukocyte count and NIHSS, mRS, or GCS. Logistic regression analysis was used to investigate the risk factors of brain herniation and death. Receiver operating characteristic (ROC) curve was performed to find the cutoff value to predict brain herniation and death. All analyses were 2‐tailed, and *P* < .05 was considered statistically significant.

## 3 RESULTS

### 3.1 Baseline characteristics

**Figure 1:**
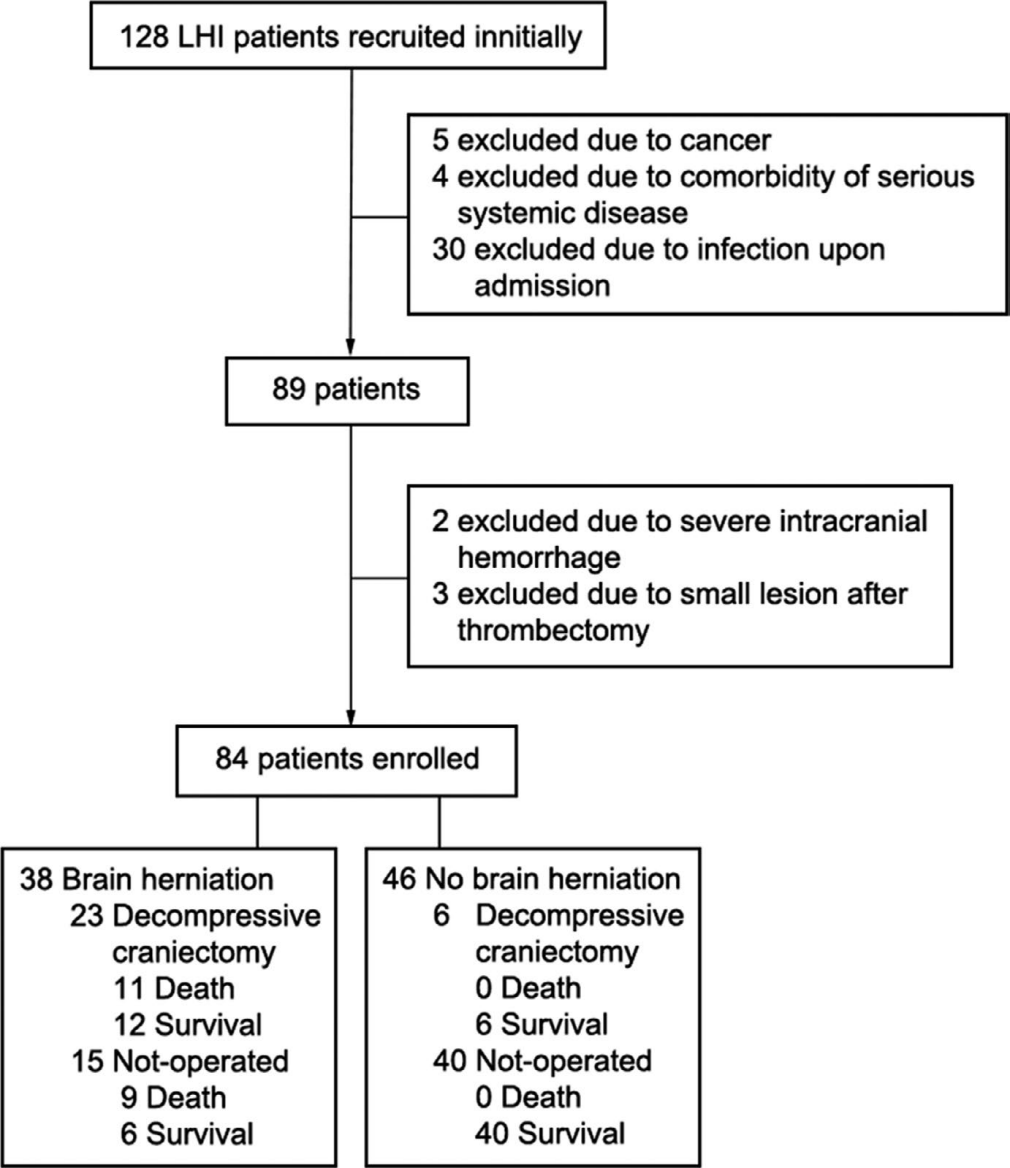
Patient enrollment flow chart

A total of 84 LHI patients (58 males and 26 females) were included in our study (Figure 1), with a mean age of 61.8 ± 14.7 years and mean NIHSS of 18.6 ± 6.2 upon admission. The patients’ baseline characteristics are described in Table 1. Among the 84 LHI patients, 38 patients developed brain herniation within 1‐6 days, and 20 patients died. The median mRS of the patients at 6 months poststroke was 5 (IQR: 4‐6).

**Table 1:** Baseline characteristics of included LHI patients

	Overall, N = 84 (%)
Age (y), mean ± SD	61.8 ± 14.1
Sex (Male/Female)	58/26
Mean arterial Pressure (mmHg), mean ± SD	103.4 ± 14.9
NIHSS, mean ± SD	18.6 ± 6.2
GCS, median (IQR)	9 (6‐10)
Body Temperature (℃), median (IQR)	36.8 (36.5‐37.3)
Hypertension	35 (41.7%)
Diabetes	17 (20.2%)
Hyperlipidemia	9 (10.7%)
Stroke history	15 (17.9%)
Coronary heart disease	9 (10.7%)
Atrial fibrillation	29 (34.5%)
Heart failure	17 (20.2%)
Smoking	43 (51.2%)
Drinking alcohol	37 (44.0%)
Family history of stroke	15 (17.9%)
TOAST classification	
Large artery atherosclerosis	50 (59.5%)
Cardiac embolism	30 (35.7%)
Others	4 (4.8%)
Postinfarct hemorrhage	8 (9.5%)
Infarction volume on D2 (cm^3^), mean ± SD	172.7 ± 102.6
Edema volume on D2 (cm^3^), median (IQR)	43.4 (22.7‐97.5)
Intravenous thrombolysis	13 (15.5%)
Intravascular treatment	5 (6.0%)
Mechanical ventilation	46 (54.8%)
Decompressive craniectomy	29 (34.5%)
mRS at 6 month, median (IQR)	5 (4‐6)

Note:

The continuous data were described as mean ± SD for normally distributed data and as median/IQR for non‐normally distributed data. Categorical data were shown as N (%).

Subgroup analysis revealed that patients with brain herniation were younger (57.6 ± 13.6 vs 65.3 ± 13.7 years old), presented with lower initial GCS score (median: 7 vs 9, *P* < .01), larger cerebral infarction, and more severe brain edema compared to those without brain herniation (*P* < .01, Table 2). Patients that died had higher NIHSS (21.4 ± 6.5 vs 17.7 ± 5.9) and lower GCS scores (median: 6 vs 9), higher incidence of coronary heart disease upon admission (25.0% vs 6.3%), more often received intravenous thrombolysis (30.0% vs 10.9%) or decompressive craniectomy (55.0% vs 28.1%, *P* < .05), and exhibited larger infarcts and more severe brain edema compared to the survivors (*P* < .01, Table 3).

**Table 2:** Clinical characteristics of LHI patients with and without brain herniation

	Brain herniation, N (%)		
	No (N = 46)	Yes (N = 38)	*P value*
Age (y), mean ± SD	65.3 ± 13.7	57.6 ± 13.6	0.012*
Sex (Male/Female)	31/15	27/11	0.718
Mean Arterial Pressure(mmHg), mean ± SD	105.0 ± 15.6	101.6 ± 14.1	0.294
NIHSS, mean ± SD	18.4 ± 6.0	18.9 ± 6.6	0.706
GCS, median/IQR	9 (8‐11)	7 (5‐9)	<0.01^**^
Temperature (℃),median/IQR	36.9 (36.5‐37.3)	36.7 (36.4‐37.3)	0.454
Hypertension	21 (45.7%)	14 (36.8%)	0.415
Diabetes	8 (17.4%)	9 (23.7%)	0.475
Hyperlipidemia	7 (15.2%)	2 (5.3%)	0.142
Stroke history	11 (23.9%)	4 (10.5%)	0.111
Coronary heart disease	4 (8.7%)	5 (13.2%)	0.51
Atrial fibrillation	16 (34.8%)	13 (34.2%)	0.956
Heart failure	12 (26.1%)	5 (13.2%)	0.142
Smoking	20 (43.5%)	23 (60.5%)	0.12
Drinking alcohol	18 (39.1%)	19 (50.0%)	0.318
Family history of stroke	8 (17.4%)	7 (18.4%)	0.902
TOAST classification			
Large artery atherosclerosis	28 (60.9%)	22 (57.9%)	
Cardiac embolism	16 (34.8%)	14 (36.8%)	
Others	2 (4.3%)	2 (5.3%)	0.955
Postinfarct hemorrhage	6 (13.0%)	2 (5.3%)	0.227
Infarction volume on D2 (cm^3^), mean ± SD	122.9 ± 79.2	227.6 ± 98.2	<0.01^**^
Edema volume on D2 (cm^3^), median (IQR)	31.0 (19.9‐54.3)	94.2 (38.8‐115.7)	<0.01^**^
Intravenous thrombolysis	4 (8.7%)	9 (23.7%)	0.059
Intravascular treatment	4 (8.7%)	1 (2.6%)	0.242
Mechanical ventilation	18 (39.1%)	28 (73.7%)	<0.01^**^
Decompressive craniectomy	6 (13.0%)	23 (60.5%)	<0.01^**^
mRS at 6 month (median/IQR)	5 (3‐5)	6 (5‐6)	<0.01^**^

Note

The continuous data were described as mean ± SD for normally distributed data and as median/IQR for non‐normally distributed data. Categorical data were shown as N (%). The P values were obtained from Student's t‐test, Mann‐Whitney U‐test or chi‐square test, when appropriate.

* *P* < .05, ^**^ *P* < .01.

**Table 3:** Clinical characteristics of LHI patients that survived or succumbed to brain herniation

	Death, N (%)		
	No (N = 64)	Yes (N = 20)	*P value*
Age (y), mean ± SD	62.1 ± 14.5	61.0 ± 13.0	0.75
Sex (Male/Female)	44/20	14/6	0.916
Mean arterial pressure (mmHg), mean ± SD	104.3 ± 14.5	100.9 ± 14.7	0.376
NIHSS, mean ± SD	17.7 ± 5.9	21.4 ± 6.5	0.02*
GCS, median/IQR	9 (7‐11)	6 (5‐8.8)	<0.01^**^
Temperature (℃), median/IQR	36.8 (36.5‐37.3)	36.7(36.3‐37.3)	0.534
Hypertension	23 (35.9%)	12 (60.0%)	0.057
Diabetes	11 (17.2%)	6 (30.0%)	0.213
Hyperlipidemia	8 (12.5%)	1 (5.0%)	0.344
Stroke history	12 (18.8%)	3 (15.0%)	0.702
Coronary heart disease	4 (6.3%)	5 (25.0%)	0.018*
Atrial fibrillation	20 (31.3%)	9 (45.0%)	0.259
Heart failure	13 (20.3%)	4 (20.0%)	0.976
Smoking	32 (50.0%)	11 (55.0%)	0.696
Drinking alcohol	28 (43.8%)	9 (45.0%)	0.922
Family history of stroke	12 (18.8%)	3 (15.0%)	0.702
TOAST classification			
Large artery atherosclerosis	39 (60.9%)	11 (55.0%)	
Cardiac embolism	21 (32.8%)	9 (45.0%)	
Others	4 (6.3%)	<0.001	0.373
Postinfarct hemorrhage	7 (10.9%)	1 (5.0%)	0.43
Infarction volume on D2 (cm^3^), mean ± SD	152.6 ± 87.1	234.4 ± 123.8	<0.01^**^
Edema volume on D2 (cm^3^), median (IQR)	43.3 (23.4‐90.2)	64.7 (18.2‐113.9)	<0.01^**^
Intravenous thrombolysis	7 (10.9%)	6 (30.0%)	0.04*
Intravascular treatment	4 (6.3%)	1 (5.0%)	0.837
Mechanical ventilation	32 (50.0%)	14 (70.0%)	0.117
Decompressive craniectomy	18 (28.1%)	11 (55.0%)	0.027*
mRS, median/IQR	5 (3‐5)	6 (6‐6)	<0.01^**^

Note:

The continuous data were described as mean ± SD for normally distributed data and as median/IQR for non‐normally distributed data. Categorical data were shown as N (%). The P values were obtained from Student's t‐test, Mann‐Whitney U‐test or chi‐square test, when appropriate.

* *P* < .05, ^**^ *P* < .01.

### 3.2 Leukocyte dynamics in LHI patients

**Figure 2:**
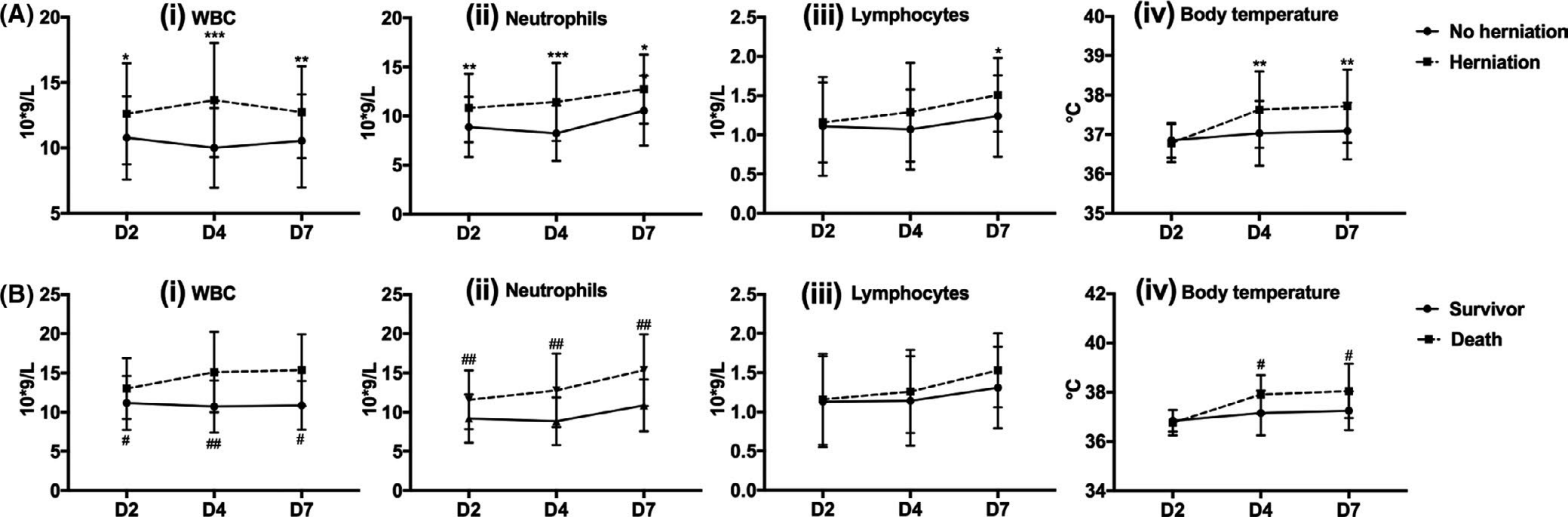
Dynamics of peripheral leukocytes and body temperature in different groups of LHI patients. *^/#^ *P* < .05, **^/##^ *P* < .01, ***P < .001

Compared to the patients with benign courses, patients developing herniation or dying from stroke showed higher WBC and neutrophil counts throughout the first week poststroke, as well as higher body temperature on D4 and D7 (*P* < .05, Figure 2). No significant difference in percentages of neutrophils/lymphocytes or NLR was found between groups (Figure S1). ΔWBC and Δneutrophil counts are presented in Figure S2. Repeated measures ANOVA revealed significant group‐time effects between WBC/neutrophil and death (*P* < .01 and *P* < .05, respectively), but not between WBC/neutrophil and brain herniation (*P* > .05). NSE level was higher in the herniation group [33.9 (19.7‐58.3) ng/mL vs 22.0 (15.1‐39.9) ng/mL, *P* < .05], but no statistically significant differences in PCT or CRP levels were observed between groups.

### 3.3 D2 WBC and neutrophil counts relate to volumes of brain infarct and edema

**Figure 3:**
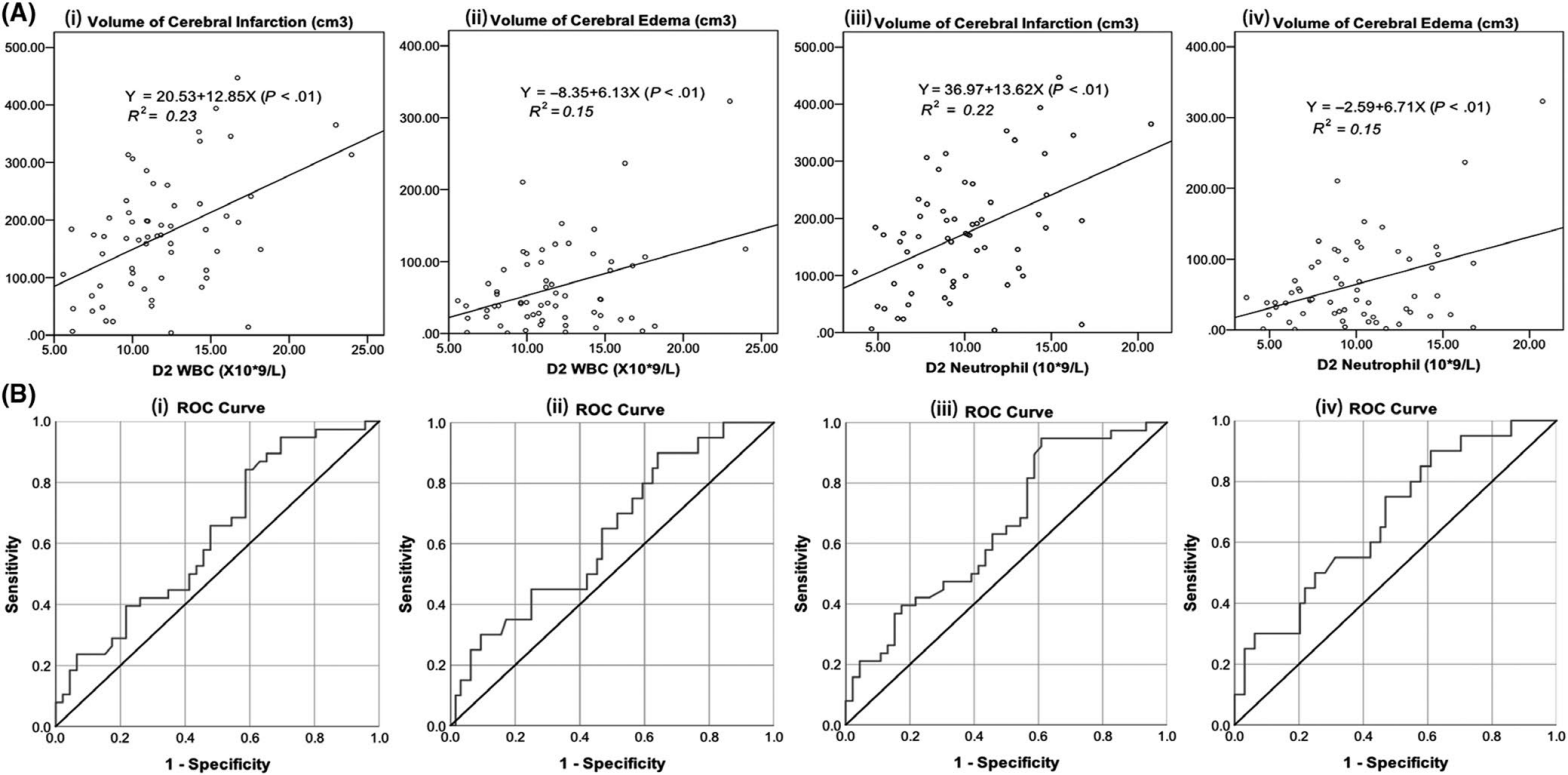
(A) Linear fitting of WBC and neutrophil counts on D2 in relation to volumes of cerebral infarction and edema in LHI patients; (B) ROC curves of WBC and neutrophil counts on D2 to predict brain herniation (i and iii, respectively) and death (ii and iv, respectively)

Pearson's correlation analysis showed that both WBC and neutrophil counts on D2 positively correlated with cerebral infarct (*R^2^* = 0.23 and *R^2^* = 0.22, respectively, *P* < .01) and edema volumes (both *R^2^* = 0.15, *P* < .01; Figure 3A i‐iv). None of the other parameters like lymphocyte count, neutrophil‐lymphocyte ratio (NLR), or NSE level was significantly correlated with the volume of cerebral infarct or edema (*P* > .05). Spearman analysis revealed that D2 neutrophil count negatively correlated with D2 GCS (ρ = −0.234, *P* < .05), but neither with NIHSS nor mRS (*P* > .05).

### 3.4 D2 neutrophil count relates to fatal outcome of LHI patients

Univariate logistic regression revealed that both WBC and neutrophil counts on D2 were significantly related to brain herniation [odds ratio (OR) = 1.166, 95% CI: 1.019‐1.334, *P* = .025; OR = 1.201, 95% CI: 1.041‐1.386, *P* = .012, respectively], and D2 neutrophil count significantly related to death (OR = 1.233, 95% CI: 1.053‐1.444, *P* = .009).

For WBC, the area under its ROC curve of D2 WBC count to predict brain herniation is 0.626 (*P* = .047, 95% CI: 0.507‐0.746) with the Youden Index of 9.75 × 10^9^ (sensitivity: 84.2%, specificity: 41.3%), and the area under its ROC curve to predict death is 0.635 (*P* = .070, 95% CI: 0.499‐0.770) (Figure 3B i, ii). Patients with D2 WBC > 9.75 × 10^9^/L had a 3.8‐fold risk of brain herniation compared to patients with D2 WBC ≤ 9.75 × 10^9^/L (95% CI: 1.3‐10.7, *P* = .011). Increased Δ_2‐4_ WBC count was associated with brain herniation (odds ratio = 2.9, 95% CI: 1.1‐7.5, *P* < .05), but not death (*P* > .05).

For D2 neutrophil count, the area under its ROC curve to predict brain herniation was 0.654 (*P* = .016, 95% CI: 0.537‐0.770) with the Youden Index of 7.14 × 10^9^/L (sensitivity: 93.1%, specificity: 37.5%), and the area under its ROC curve to predict death was 0.678 (*P* = .017, 95% CI: 0.548‐0.809) with the Youden Index of 7.79 × 10^9^/L (sensitivity: 90.0%, specificity: 39.1%) (Figure 3B iii, iv). Patients with D2 neutrophil > 7.14 × 10^9^/L had a 7.5‐fold risk of brain herniation compared to patients with D2 neutrophil ≤ 7.14 × 10^9^/L (95% CI: 2.0‐28.1, *P* = .001). Patients with D2 neutrophil > 7.79 × 10^9^/L had a 5.8‐fold risk of death compared to patients with D2 neutrophil ≤ 7.79 × 10^9^/L (95% CI: 1.2‐27.0, *P* = .015). Increased Δ_2‐4_ neutrophil count was associated with brain herniation (odds ratio = 2.7, 95% CI: 1.0‐6.7, *P* < .05), but not death (*P* = .52).

## 4 DISCUSSION

Our study found that LHI patients developing brain herniation or succumbing to stroke showed higher WBC and neutrophils counts within the first week after stroke. Neutrophil count on D2 after stroke significantly related to infarct and edema volumes as well as GCS, and also related to fatal outcome (brain herniation and death).

Animal studies revealed that neutrophils rapidly increase within a few hours after stroke and peak at 24‐48h before gradually returning to baseline.^12^, ^13^, ^14^ They contribute to the disruption of blood‐brain barrier and aggravate ischemic injury and edema by releasing reactive oxygen species, proteases (metalloproteinases, elastase, cathepsin G, etc), cytokines (eg, IL‐6, IL‐8, TNF‐α), and chemokines (CCL2, CCL3, CCL5, etc).^13^, ^15^ They also play a multifaceted role in various stroke‐related aspects such as thrombosis and poststroke infection.^12^, ^13^ Our results showed peripheral neutrophil count peaked on D2 after stroke and then gradually decreased on D4 in LHI patients without brain herniation. Similar changes were observed in stroke survivors. In contrast, neutrophil count further increased until D4 in patients with brain herniation. Hence, it may be proposed that neutrophil infiltration is more severe and persists for a longer time in malignant cerebral edema or prior to death (Figure S3).

In our study, D7 neutrophil counts peaked across groups, which might be related to the frequent poststroke pneumonia. Poststroke pneumonia might be a respiratory syndrome due to complex bacterial, chemical, and immunological factors, and could not be prevented by prophylactic antibiosis alone, even for patients at high aspiration risk.^16^ All patients with known infection upon admission were excluded in our study. Moreover, body temperature on D2, as well as the levels of PCT and CRP, was comparable between groups. Thus, potential effects of early infection on D2 neutrophil count are unlikely. However, patients that developed pneumonia thereafter were not excluded due to high incidence in each group (>90%), and exclusion of such patients would therefore dramatically decrease sample size and might further cause a selection bias.

Our results provide preliminary evidence for a linear relationship between neutrophil count and infarct/edema volumes, which has not been established previously. Furthermore, neutrophil count was found to significantly relate to brain herniation and death after LHI. Various imaging modalities such as perfusion CT, PET, and MRI within 6‐24h also have been reported to reliably predict a malignant LHI course mainly through evaluating infarct and edema volumes.^17^, ^18^, ^19^ However, availability and feasibility of these imaging modalities are limited due to practical reasons early after symptom onset in many stroke units. Other biomarkers such as NLR, PCT, and NSE have been suggested to predict the severity of ischemic stroke,^20^, ^21^, ^22^ but none of them was found to relate to infarct or edema volumes in this study. Hence, neutrophil count could be of clinical use as a simple yet sensitive indicator for LHI patients at risk of fatal outcome and would be helpful to indicate the necessity and timing of specific imaging and subsequent additional therapeutic measures such as careful dehydration and decompressive craniectomy to reduce intracranial pressure. However, specificity of D2 neutrophil count as a predictor of fatal outcome was poor (<40%), indicating that other risk factors also contribute to fatal outcome after LHI.

Our study has some limitations. First, the modest sample size limits the data interpretation. Second, other causes of peripheral leukocytosis cannot be completely excluded despite controlling for signs of infection upon admission. Third, only limited data were obtained in the hyper‐acute stage after stroke because many patients arrived relatively late, and any analysis at very early time points is therefore not possible. Fourth, longitudinal imaging was not performed on D4 and D7 in order to reduce frequent transportation of those critically ill patients. Therefore, it is unknown whether the increase of WBC and neutrophil counts on D7 relates primarily to the growth of infarct or edema, or the frequently observed pneumonia.

In conclusion, our study provides preliminary evidence that increased peripheral neutrophil count early after LHI relates to infarct and edema volumes, and can indicate fatal outcome of LHI patients, thus may help guiding acute management of LHI such as reduction of intracranial pressure. As a promising target in stroke therapy, neutrophils may also be used to identify patients that could benefit from adjunctive antiinflammatory therapies.^15^ Future studies with larger numbers of patients are required to verify this hypothesis and shall also focus specifically on earlier time points after stroke (≤24h).

## CONFLICT OF INTEREST

The authors declare no conflict of interest.

## Supporting information

Supplementary Material

## References

[1] Frank JI . Large hemispheric infarction, deterioration, and intracranial pressure. Neurology. 1995;45:1286‐1290. 7617183 10.1212/wnl.45.7.1286

[2] MT1, Bösel J, Rhoney DH, , et al. Evidence‐based guidelines for the management of large hemispheric infarction: a statement for health care professionals from the Neurocritical Care Society and the German Society for Neuro‐intensive Care and Emergency Medicine. Neurocrit Care. 2015;22:146‐164. 25605626 10.1007/s12028-014-0085-6

[3] Jüttler E , Unterberg A , Woitzik J , et al. Hemicraniectomy in older patients with extensive middle‐cerebral‐artery stroke. N Engl J Med. 2014;370:1091‐1100. 24645942 10.1056/NEJMoa1311367

[4] Zhao J , Su YY , Zhang Y , et al. Decompressive hemicraniectomy in malignant middle cerebral artery infarct: a randomized controlled trial enrolling patients up to 80 years old. Neurocrit Care. 2012;17:161‐171. 22528280 10.1007/s12028-012-9703-3

[5] Iadecola C , Anrather J . The immunology of stroke: from mechanisms to translation. Nat Med. 2012;17:796‐808. 10.1038/nm.2399 PMC3137275 21738161

[6] Frieler RA , Chung Y , Ahlers CG , et al. Genetic neutrophil deficiency ameliorates cerebral ischemia‐reperfusion injury. Exp Neurol. 2017;298:104‐111. 28865993 10.1016/j.expneurol.2017.08.016 PMC5658240

[7] Hermann DM , Kleinschnitz C , Gunzer M . Implications of polymorphonuclear neutrophils for ischemic stroke and intracerebral hemorrhage: Predictive value, pathophysiological consequences and utility as therapeutic target. J Neuroimmunol. 2018;321:138‐143. 29729895 10.1016/j.jneuroim.2018.04.015

[8] Price CJ , Menon DK , Peters AM , et al. Cerebral neutrophil recruitment, histology, and outcome in acute ischemic stroke: an imaging‐based study. Stroke. 2004;35:1659‐1664. 15155970 10.1161/01.STR.0000130592.71028.92

[9] Jauch EC , Saver JL , Adams HP Jr , et al. Guidelines for the early management of patients with acute ischemic stroke: a guideline for healthcare professionals from the American Heart Association/American Stroke Association. Stroke. 2013;44:870‐947. 23370205 10.1161/STR.0b013e318284056a

[10] Gerriets T , Stolz E , Walberer M , et al. Noninvasive quantification of brain edema and the space‐occupying effect in rat stroke models using magnetic resonance imaging. Stroke. 2004;35:566‐571. 14739415 10.1161/01.STR.0000113692.38574.57

[11] Adams HP Jr , Bendixen BH , Kappelle LJ , et al. Classification of subtype of acute ischemic stroke. Definitions for use in a multicenter clinical trial. TOAST. Trial of Org 10172 in Acute Stroke Treatment. Stroke. 1993;24:35‐41. 7678184 10.1161/01.str.24.1.35

[12] Ruhnau J , Schulze J , Dressel A , Vogelgesang A . Thrombosis, neuroinflammation, and post stroke infection: the multifaceted role of neutrophils in stroke. J Immunol Res. 2017;2017:5140679. 28331857 10.1155/2017/5140679 PMC5346374

[13] Chu HX , Kim HA , Lee S , et al. Immune cell infiltration in malignant middle cerebral artery infarction: comparison with transient cerebral ischemia. J Cereb Blood Flow Metab. 2014;34:450‐459. 24326388 10.1038/jcbfm.2013.217 PMC3948121

[14] Möller K , Boltze J , Pösel C , et al. Sterile inflammation after permanent distal MCA occlusion in hypertensive rats. J Cereb Blood Flow Metab. 2014;34:307‐315. 24220169 10.1038/jcbfm.2013.199 PMC3915208

[15] Jickling GC , Liu D , Ander BP , et al. Targeting neutrophils in ischemic stroke: translational insights from experimental studies. J Cereb Blood Flow Metab. 2015;35:888‐901. 25806703 10.1038/jcbfm.2015.45 PMC4640255

[16] Kalra L , Irshad S , Hodsoll J , et al. Prophylactic antibiotics after acute stroke for reducing pneumonia in patients with dysphagia (STROKE‐INF): a prospective, cluster‐randomized, open‐label, masked endpoint, controlled clinical trial. Lancet. 2015;386:1835‐1844. 26343840 10.1016/S0140-6736(15)00126-9

[17] Dohmen C , Bosche B , Graf R , et al. Prediction of malignant course in MCA infarction by PET and microdialysis. Stroke. 2003;34:2152‐2158. 12881606 10.1161/01.STR.0000083624.74929.32

[18] Minnerup J , Wersching H , Ringelstein EB , et al. Prediction of malignant middle cerebral artery infarction using computed tomography‐based intracranial volume reserve measurements. Stroke. 2011;42:3403‐3409. 21903965 10.1161/STROKEAHA.111.619734

[19] Thomalla G , Hartmann F , Juettler E , et al. Prediction of malignant middle cerebral artery infarction by magnetic resonance imaging within 6 hours of symptom onset: A prospective multicenter observational study. Ann Neurol. 2010;68:435‐445. 20865766 10.1002/ana.22125

[20] Goyal N , Tsivgoulis G , Chang JJ , et al. Admission neutrophil‐to‐lymphocyte ratio as a prognostic biomarker of outcomes in large vessel occlusion strokes. Stroke. 2018;49:1985‐1987. 30002151 10.1161/STROKEAHA.118.021477

[21] Zhang Y , Liu G , Wang Y , et al. Procalcitonin as a biomarker for malignant cerebral edema in massive cerebral infarction. Sci Rep. 2018;8:993. 29343753 10.1038/s41598-018-19267-4 PMC5772664

[22] Haupt WF , Chopan G , Sobesky J , Liu WC , Dohmen C . Prognostic value of somatosensory evoked potentials, neuron‐specific enolase, and S100 for short‐term outcome in ischemic stroke. J Neurophysiol. 2016;115:1273‐1278. 26745251 10.1152/jn.01012.2015 PMC4808083

