# Supplementary material for: Early neutrophil count relates to infarct size and fatal outcome after large hemispheric infarction
Source: CNS Neurosci Ther. 2020 May 6;26(8):829–36. doi: 10.1111/cns.13381 (PMC7366744; doi:10.1111/cns.13381)

**SUPPLEMENTAL MATERIAL**

**Supplemental Table 1. Baseline characteristics of included LHI patients.**

|  | Overall, N=84 (%) |
| --- | --- |
| Systolic Blood Pressure (mmHg), mean±SD | 147.0±24.2 |
| Diastolic Blood Pressure (mmHg), mean±SD | 81.5±13.4 |
| Symptoms within 48h | |
| Vomit | 25 (29.8%) |
| Headache | 13 (15.5%) |
| Reduced consciousness | 62 (73.8%) |
| Gaze palsy | 56 (66.7%) |
| Progressive worsening | 58 (69.0%) |

Note: The continuous data were described as mean ± SD for normally distributed data. Categorical data was shown as N (%).

**Supplemental Table 2. Clinical characteristics of LHI patients with and without brain herniation.**

|  | Brain herniation, N (%) | | |
| --- | --- | --- | --- |
|  | No (N=46) | Yes (N=38) | *P value* |
| Systolic Blood Pressure (mmHg), mean±SD | 148.2±26.0 | 82.8±13.5 | 0.63 |
| Diastolic Blood Pressure(mmHg), mean±SD | 82.8±13.5 | 79.9±13.2 | 0.327 |
| Symptoms within 48h | | | |
| Vomit | 9 (19.6%) | 16 (42.1%) | 0.025^*^ |
| Headache | 6 (13.0%) | 7 (18.4%) | 0.498 |
| Reduced consciousness | 32 (69.6%) | 30 (78.9%) | 0.33 |
| Gaze palsy | 29 (63.0%) | 27 (71.1%) | 0.438 |
| Progressive worsening | 25 (54.3%) | 33 (86.8%) | <0.01^**^ |

Note: The continuous data were described as mean ± SD for normally distributed data. Categorical data was shown as N (%). The P values were obtained from the Student’s t or chi-square tests, when appropriate. ^*^P<0.05, ^**^P<0.01.

**Supplemental Table 3.** **Clinical characteristics of LHI patients that survived or succumbed to brain herniation.**

|  | Death, N (%) | | |
| --- | --- | --- | --- |
|  | No (N=64) | Yes (N=20) | *P value* |
| Systolic Blood Pressure (mmHg),mean±SD | 147.6±25.0 | 144.9±22.0 | 0.661 |
| Diastolic Blood Pressure (mmHg),mean±SD | 82.3±13.1 | 78.9±14.3 | 0.322 |
| Symptoms within 48h | | | |
| Vomit | 13 (20.3%) | 12 (60.0%) | <0.01^**^ |
| Headache | 11 (17.2%) | 2 (10.0%) | 0.438 |
| Consciousness disorder | 45 (70.3%) | 17 (85.0%) | 0.192 |
| Gaze palsy | 44 (68.8%) | 12 (60.0%) | 0.469 |
| Progressive worsening | 40 (62.5%) | 18 (90.0%) | 0.02^*^ |

Note: The continuous data were described as mean ± SD for normally distributed data. Categorical data was shown as N (%). The P values were obtained from the Student’s t or chi-square tests, when appropriate. ^*^P<0.05, ^**^P<0.01.

**Supplemental Figure Legends**

**Supplemental Figure II.** ΔWBC and Δneutrophils within the first week after stroke in different groups. *P<0.05, herniation vs. no herniation group; ^#/##^P<0.05/<0.01, survivor vs. death group.

**Supplemental Figure III.** Conceptual hypothesis of neutrophil count changes after ischemic stroke in patients with or without brain herniation and the death.

**Supplemental Figure I**


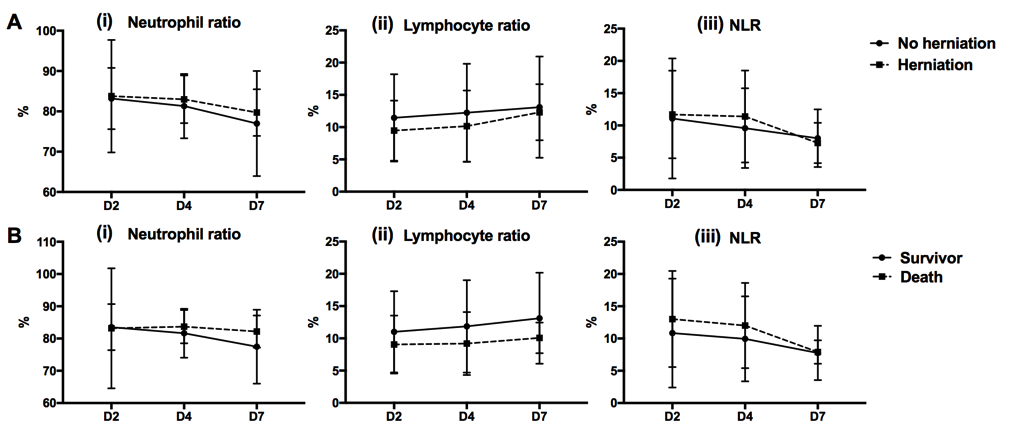


**Supplemental Figure II**


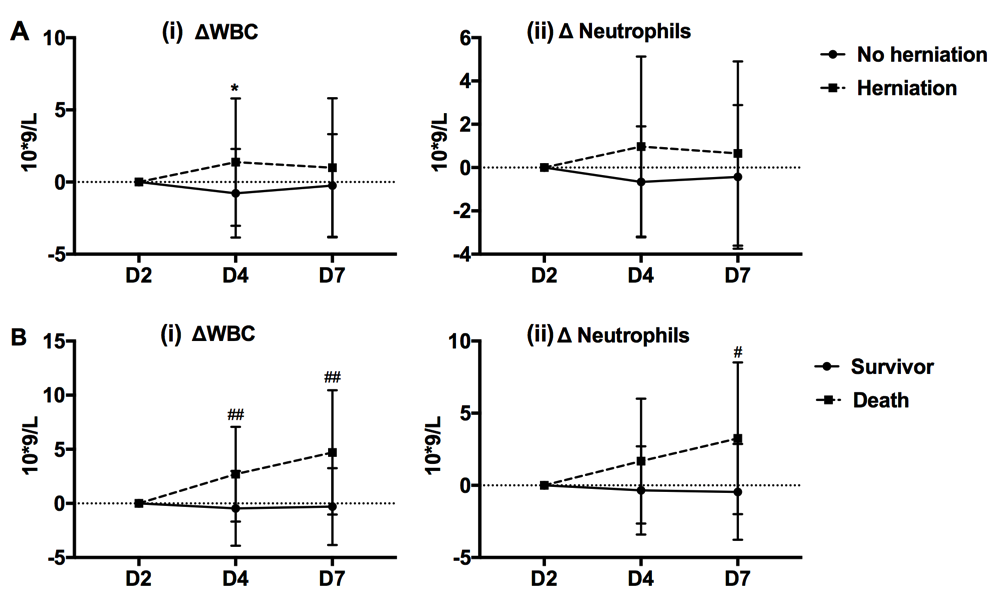


**Supplemental Figure III**


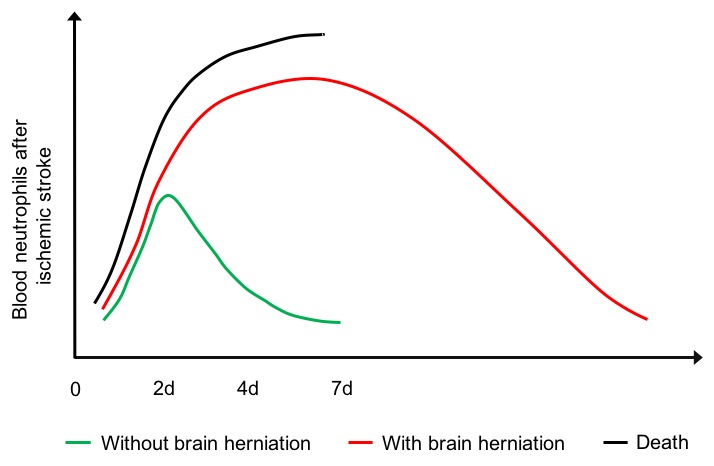

Supplement: Supplementary file 1 — Supplementary Material [file CNS-26-829-s001.docx]
